# Abnormal Liver Tests during Hospitalization Predict Mortality in Patients with COVID-19: A Multicenter Study from South America

**DOI:** 10.1155/2021/1622533

**Published:** 2021-10-05

**Authors:** Domingo Balderramo, Angelo Z. Mattos, Victoria Mulqui, Talita Chiesa, Zuly Plácido-Damián, Jaysoom Abarca, Andrea Bolomo, Yanina Carlino, Isadora Z. Bombassaro, Denusa Wiltgen, Laura Tenorio Castillo, Karina Díaz, Johana Acuña, Estela Manero, Jhon Prieto, Enrique Carrera, Javier Díaz-Ferrer, Jose D. Debes

**Affiliations:** ^1^Gastroenterology Department, Hospital Privado Universitario de Córdoba, Instituto Universitario de Ciencias Biomédicas de Córdoba, Córdoba, Argentina; ^2^Graduate Program in Medicine: Hepatology, Federal University of Health Sciences of Porto Alegre, Porto Alegre, Brazil; ^3^Gastroenterology and Hepatology Unit, Irmandade Santa Casa de Misericórdia de Porto Alegre, Porto Alegre, Brazil; ^4^Internal Medicine Department, Hospital Pablo Soria, San Salvador de Jujuy, Argentina; ^5^Gastroenterology Department, Hospital Nacional Edgardo Rebagliati Martins-Essalud, School of Medicine, Universidad Nacional Mayor de San Marcos, Lima, Peru; ^6^Department of Gastroenterology, Hospital Especialidades Eugenio Espejo, Universidad San Francisco de Quito, Quito, Ecuador; ^7^Internal Medicine Unit, Irmandade Santa Casa de Misericórdia de Porto Alegre, Porto Alegre, Brazil; ^8^Centro de Enfermedades Hepaticas y Digestivas, Bogota, Colombia; ^9^Department of Medicine, University of Minnesota, Minneapolis, USA

## Abstract

**Background:**

The role of liver function tests (LFT) as prognostic factors in patients admitted with COVID-19 has not been fully investigated, particularly outside resource-rich countries. We aimed at evaluating the prognostic value of abnormal LFT on admission and during hospitalization of patients with COVID-19.

**Methods:**

We performed a retrospective study that included 298 adult patients hospitalized for COVID-19, between 05/2020 and 02/2021, in 6 hospitals from 5 countries in South America. We analyzed demographic and comorbid variables and laboratory tests on admission and during hospitalization. LFT over twice the upper limit of normal (ALEx2) were also evaluated in relation to a variety of factors on admission and during hospitalization. De novo-ALEx2 was defined as the presence of ALEx2 at one week of hospitalization in patients without ALEx2 on admission. Patients were followed until hospital discharge or death. Multivariable analysis was used to evaluate the association between ALEx2 on admission and during hospitalization and mortality.

**Results:**

Of the total of 298 patients, 60% were male, with a mean age of 60 years, and 74% of patients had at least one comorbidity. Of those, 137 (46%) patients were transferred to the intensive care unit and 66 (22.1%) patients died during hospitalization. ALEx2 on admission was present in 87 (29.2%) patients and was found to be independently associated with 1-week mortality (odds ratio (OR) = 3.55; 95% confidence interval (95%CI) 1.05–12.05). Moreover, 84 (39.8%) out of 211 patients without ALEx2 at admission developed de novo-ALEx2, which was independently associated with mortality during second week of hospitalization (OR = 6.09; 95%CI 1.28–29) and overall mortality (OR = 2.93, 95%CI 1.05–8.19).

**Conclusions:**

A moderate elevation of LFT during admission was associated with a poor short-term prognosis in patients hospitalized with COVID-19. In addition, moderate elevation of LFT at one week of hospitalization was an independent risk factor for overall mortality in these patients.

## 1. Introduction

Severe acute respiratory syndrome coronavirus 2 (SARS-CoV-2) infection causes coronavirus disease 2019 (COVID-19), which is a rapidly emerging disease that has led to a pandemic of proportions never before seen in modern times. Furthermore, the global mortality associated with the virus has advanced at an unprecedented rate in different regions of the world. Multiple studies have identified several prognosis factors during COVID-19, including age greater than 60 years and comorbidities such as diabetes, cardiovascular disease, or obesity [1, 2]. However, scarce prognostic information is available from populations from South America, a region experiencing a significant impact related to COVID-19 pandemic [3, 4].

SARS-CoV-2 infection presents a significant heterogeneity in its clinical course, ranging from asymptomatic presentations to life-threatening disease such as acute respiratory distress syndrome or multiple organ failure [5]. A great number of infected patients, mainly those critically ill with SARS-CoV-2, present gastrointestinal manifestations, particularly acute alteration of liver function [6, 7]. Several studies have shown that patients with COVID-19 have evidence of liver damage on admission for hospitalization (ranging from 14% to 53%), expressed mainly by abnormal levels of liver transaminases but also by slightly elevated bilirubin levels [8]. In cases of severe COVID-19, the incidence of liver injury can reach 93%, indicating a possible association between COVID-19-related liver disease and mortality [9]. However, there is little information available related to modifications of liver function tests (LFT) during hospitalization and their role as a marker of severity in patients with severe COVID-19 [10].

The aim of the present study was to assess the impact of SARS-CoV-2 infection on LFT on admission and during hospitalization, as well as the prognostic role of abnormal LFT in hospitalized patients with COVID-19 in South America.

## 2. Methods

A multicenter and retrospective study was performed in order to describe adult patients hospitalized for COVID-19 between 05/2020 and 02/2021 in 6 hospitals from Argentina, Ecuador, Brazil, Peru, and Colombia. Approval was obtained from each institutional review board from all participating centers. A waiver of informed consent was granted for this study, considering its retrospective design.

### 2.1. Study Population

Patients were included if they were ≥18 years old at the time of hospitalization and were admitted with SARS-CoV-2 infection confirmed by the real-time polymerase chain reaction (RT-PCR) according to the site-specific protocol and if LFT were evaluated on admission. Patients admitted for other causes, but who were then later diagnosed with SARS-CoV-2 infection during hospitalization or had asymptomatic presentations with positive RT-PCR testing, were excluded. Patients who were pregnant at the time of diagnosis and patients with incomplete medical records were also excluded. All patients were followed until discharge or death.

### 2.2. Variables

Demographics, clinical information, routine and inflammatory laboratory markers, comorbidities, and radiological studies were evaluated at the moment of admission. Etiology of those patients with diagnosis of liver disease was assessed according to history or prior virologic markers. Also, autoimmune liver diseases were excluded according to history or evaluation of autoantibodies during admission [11–13]. Information about medications taken in the 10 days previous to admission was also analyzed.

During hospitalization, data related to admission to intensive care unit (ICU), use of vasopressor drugs (use of vasopressors >12 hours), requirement for mechanical ventilation, length of stay in ICU, length of hospitalization, and mortality were evaluated. LFT, blood count, kidney function tests, and inflammatory markers at 1 week of hospitalization were also evaluated when available.

To assess the impact of abnormal LFT on admission, patients included in the study were categorized into two groups, according to the presence or not of a moderate abnormal value of any LFT >2 times over the upper limit of normal (ULN) on admission (group ALEx2). Thus, we defined ALEx2 as the elevation of at least one of the following: total bilirubin (TBil), alanine aminotransferase (ALT), aspartate aminotransferase (AST), gamma-glutamyl transferase (GGT), or alkaline phosphatase (ALP) values to levels greater than twice the ULN value. ULN was taken as the reference value from each participating center.

During hospitalization, de novo-ALEx2 was defined as the occurrence of ALEx2 at 1 week of hospitalization in patients without ALEx2 at admission. Persistent-ALEx2 was defined as the persistence of ALEx2 at 1 week of hospitalization in patients with ALEx2 at admission.

The primary endpoint evaluated was overall mortality. As admission variables may have a different impact during the course of COVID-19 hospitalization, mortality was also evaluated at 1 and 2 weeks after admission.

### 2.3. Statistical Analysis

Continuous variables were expressed as mean and standard deviation, or as median and interquartile range (IQR), according to their homogeneity. Categorical variables were expressed as number and percentage and were compared using the chi-square test or Fisher's exact test according to the expected frequencies. Continuous variables were compared with the *T* test or the Mann–Whitney test, according to their homogeneity. Values were expressed as odds ratio (OR) and a 95% confidence interval (95% CI). A multivariable logistic regression was used to evaluate the association between ALEx2 and mortality. We first fitted univariate models to evaluate crude effects on mortality related to different variables, including age, sex, comorbidities, clinical and laboratory findings on admission, and LFT at 1 week of hospitalization. We constructed final multivariable models, including variables with a *P* value <0.1 in univariate analysis, at 1 and 2 weeks and the overall mortality, respectively. A probability value < 0.05 was used to define statistical significance. The statistical analysis was performed with the statistical software IBM SPSS version 24 (SPSS inc, Armonk, NY.).

## 3. Results

### 3.1. Characteristics of the Study Population

During the study period, 337 adult inpatients with confirmed COVID-19 were identified. Of these, 39 had no data relating to LFT at admission and were excluded. Therefore, a total of 298 patients were included in the final analysis. The mean patient age was 60 ± 16 years, and 60% of these individuals were male (Table 1). In all, 74% of patients had at least one comorbidity, with cardiovascular disorders being the most common (24%), followed by obesity (19%) and diabetes (14%). Twenty-one patients (7%) had a history of chronic liver disease, among which 17 of these had nonalcoholic fatty liver disease (NAFLD), 3 had alcoholic liver disease, and 2 had untreated HCV infection (one patient also had alcoholic liver disease). Three patients (1%) had a history of cirrhosis. No patients had history of autoimmune liver disease and 11 patients had history of negative autoantibodies prior to admission. During hospitalization autoantibodies tests were performed in 6 patients and all results were negative. Table 1 describes the medications taken in the 10 days prior to admission. About half of the patients took potentially hepatotoxic medications, including antibiotics (34%), acetaminophen (16%), nonsteroidal anti-inflammatory drugs (NSAIDs, 8%), and statins (7%). The most frequent symptoms of COVID-19 during admission were dyspnea (74%), fever (65%), cough (59%), diarrhea (17%), nausea/vomiting (7%), and abdominal pain (8%). Time from symptoms onset to admission was 7 days (IQR 4–10). The most common radiological findings were bilateral pulmonary infiltrate/consolidation (83%) (Table 1).

### 3.2. Characteristics of Patients with ALEx2 at Admission

During admission, 87 (29.2%) out of 298 patients presented ALEx2. The main characteristics of patients with or without ALEx2 are described in Table 1. Patients without ALEx2 showed a higher rate of comorbidities (77% vs. 65%; *P*=0.036). No significant differences were found for any specific comorbidity, although there was a trend for a higher rate of chronic liver disease in patients with ALEx2 (11% vs. 5%; *P*=0.054). NAFLD also tended to be more frequent in patients with ALEx2 (9% vs. 4%; *P*=0.095). No association was found between ALEx2 and medications taken in the previous 10 days prior to admission. Patients with ALEx2 presented similar symptoms at admission when compared to those without ALEx2. However, the time from symptoms onset to hospitalization was longer in the group of patients with ALEx2 (7 days (IQR 5–10) vs. 6 days (IQR 3–9), *P*=0.017). Similarly, patients with ALEx2 had higher levels of D-dimer, lactate dehydrogenase, ferritin, and neutrophils/lymphocytes ratio and a lower platelet count at admission (Table 1). White blood cells (WBC) count, C-reactive protein, hemoglobin, albumin, and procalcitonin on admission were similar in both groups of patients. Concerning thoracic radiological findings at admission, bilateral infiltrate/consolidation was more frequent in patients with ALEx2 (91% vs. 80%, *P*=0.02). Liver imaging studies were available only in 34 (11.4%) patients during hospitalization (15 in the group of ALEx2 and 19 in those without ALEx2). Changes in liver homogeneity were present in 13 patients with ALEx2 and in 15 patients without ALEx2, and ascites was observed in 2 patients with ALEx2 and in one patient without ALEx2.

### 3.3. Clinical Course of COVID-19 and Admission to ICU

A total of 230 (77.2%) patients were discharged and 66 (22.1%) patients died during hospitalization, with the median time from admission to death being 17 days (IQR 11–25.8). Fifty (75.8%) out of 66 patients who died did so within 3 weeks of hospitalization, and the remaining 16 (24.2%) patients died between 25 and 59 days after admission. During their hospital stay, 137 (46%) patients were transferred to the ICU, with 65 (21.8%) requiring vasopressors. A total of 99 (33%) patients required mechanical ventilation, with a median length on mechanical ventilation of 14 days (IQR 8–19). The median length of ICU admission was 13 days (IQR 6–20.8) and the median global length of hospitalization was 13 days (IQR 7–22).

### 3.4. ALEx2 at Admission: Clinical Course during Hospitalization

The proportion of patients transferred to the ICU, requirement for vasopressors, and the need of mechanical ventilation during hospitalization were similar in those with or without ALEx2 (Table 2). Overall mortality was also similar in both groups (24 vs. 22%). However, mortality during the first week of admission was higher in patients with ALEx2 (9% vs. 3%, *P*=0.031). The median time from admission to death was shorter in patients with ALEx2 (11 days (IQR 5–18) vs. 18 days (IQR 13–30), *P*=0.034). Finally, the length of ICU hospitalization was similar in both groups, but the overall length of hospitalization tended to be longer in patients without ALEx2 (14 days (IQR 7–23.5) vs. 11 days (IQR 7–20), *P*=0.055).

### 3.5. ALEx2 during Hospitalization: Characteristics and Clinical Course

A total of 42 (48.3%) out of 87 patients with ALEx2 upon admission continued with abnormal LFT >2 times UNL (persistent-ALEx2 group). On the other hand, 84 (39.8%) out of 211 patients developed de novo-ALEx2. Patients with or without persistent-ALEx2 presented a similar need of ICU admission (50% vs. 49%, *P*=0.2), requirement for mechanical ventilation (41% vs. 51%, *P*=0.23), and use of vasopressors (29% vs. 32%, *P*=0.78). Similarly, patients with or without de novo-ALEx2 presented a similar need of ICU admission (58% vs. 42%, *P*=0.8), need for mechanical ventilation (49% vs. 48%, *P*=0.93), and use of vasopressors (28% vs. 34%, *P*=0.47). Age, sex, and comorbidities, including chronic liver disease, were similar in patient with or without de novo-ALEx2. In addition, routine and inflammatory markers such as C-reactive protein, procalcitonin, ferritin, and D-dimer on admission and at one week of hospitalization were similar in patients with or without de novo-ALEx2. The patterns of the abnormal LFT in patients with ALEx2 at admission and de novo-ALEx2 are shown in Table 3. AST and ALT were the most frequently altered LFT at both time points.

### 3.6. Predictors of COVID-19-Associated Mortality during the First and Second Weeks and Overall Hospitalization

We performed a multivariate logistic regression analysis to evaluate the role of ALEx2 (on admission and during hospitalization) as an independent factor associated with mortality during different periods of hospitalization. After including significant variables in the univariate analysis and adjusting for age, gender, comorbidities, and WBC count at admission, ALEx2 at admission was found to be independently associated with 1-week mortality (OR = 3.55; 95% CI 1.05–12.05; *P*=0.042) (Table 4). When mortality during the second week of hospitalization was evaluated and after including significant variables in the univariate analysis and adjusting for age, gender, and comorbidities, de novo-ALEx2 was identified as an independent risk factor for mortality (OR = 6.09; 95% CI 1.28–29; *P*=0.022) (Table 4). In the final model evaluating overall mortality, after including all significant variables in the univariate analysis and adjusting for age, gender, comorbidities, active smocking, and WBC count at one week of hospitalization, de novo-ALEx2 was also found to be an independent risk factor for mortality (OR = 2.93; 95% CI 1.05–8.19; *P*=0.04) (Table 4).

## 4. Discussion

In the present study, which analyzed data obtained from several hospitals in South America, we found that the moderate alteration of LFT at admission was a factor associated with a worse short-term prognosis in patients with COVID-19. In addition, we showed that the de novo alteration of LFT at one week of hospitalization was an independent risk factor for overall mortality in patients hospitalized for COVID-19.

Moderately (2–5 times the ULN) abnormal LFT at admission have been reported in 4–32% of patients hospitalized for COVID-19, and a more severe course during hospitalization has been observed in these patients [7, 9, 14–16]. In the present study ALEx2 at admission was observed in one-third of patients admitted for COVID-19, with a small proportion of these patients presenting a history of chronic liver disease. We verified that this group of patients presented a higher mortality during the first week of hospitalization than those without this biochemical alteration, an association which was independent of age and associated comorbidities including cardiovascular and chronic liver disease. Moreover, the period of time between admission and death was shorter in patients with ALEx2. However, in contrast to other studies, we detected a similar rate of overall mortality for patients with or without ALEx2 at admission [16–18]. This finding is possibly related to the influence of a higher percentage of comorbidities in the group of patients without ALEx2, which was associated with a severe course of COVID-19 in prior studies [5, 15, 16]. The observation that comorbidities were less common in patients with ALEx2 at admission in the present study reinforces their role as independent initial marker for worse short-term outcome.

A variety of factors present prior to hospitalization may be related to abnormal LFT on admission, with previous studies highlighting age, male sex, and comorbidities, including hypertension, obesity, and chronic liver disease [16, 18–20]. In our study, only male sex was associated with ALEx2 on admission, and a lower percentage of comorbidities was present in this group. Some prior studies have shown that the use of different medications before admission may be related to abnormal LFT at admission [9, 16]. However, we observed that the medication use during the 10 days prior to hospitalization was similar in patients with or without ALEx2 on admission, indicating that drug-induced liver injury may not be the main reason for abnormal LFT at admission in the present cohort [9].

We also observed that the presence of de novo-ALEx2 at one week of hospitalization was related to mortality during the second week of hospitalization and to overall mortality. A previous study that evaluated LFT during hospitalization demonstrated that patients with de novo-liver test abnormalities presented a trend for a higher requirement of ICU admission or death [21]. The evaluation of de novo-ALEx2 can distinguish patients who present normal or subtle elevation of LFT at admission with a progressive increase during hospitalization [9]. In this regard, another study showed that the peak level of ALT in patients with severe COVID-19 infection was progressively reached within 10–15 days after admission [7].

The presence of de novo-ALEx2 could be related to the combination of multiple factors including baseline comorbidities, medications prescribed during early hospitalization, or liver injury related to SARS-CoV-2-induced systemic inflammatory response [7, 21–23]. In our study, age, sex, and comorbidities were similar in patients with or without de novo-ALEx2. The inflammatory markers at admission and at one week of hospitalization were also similar in patients with or without de novo-ALEx2. The influence of drug toxicity has been evaluated in other studies, which have shown the use of lopinavir and ritonavir to be risk factors for liver test abnormalities [9, 19]. In our study, therapy carried out during hospitalization was not evaluated because of the complexity of interpretation and the lack of a uniform algorithm among participating centers. As the role of de novo-ALEx2 has not been fully evaluated as a marker of the hospitalization course, this deserves further validation in prospective studies.

Although the exact pathogenesis of liver injury in COVID-19 remains unknown [22], our findings suggest, similar to other studies, that liver enzymes abnormalities are related to SARS-CoV-2 infection itself [9]. On the one hand, hepatocytes do not express high levels of angiotensin-converting enzyme 2 receptor (ACE2), which is involved in SARS-CoV-2 cell entry, and therefore these cells are unlikely to be a target of this virus [24]. On the other hand, cholangiocytes express high levels of ACE2 and could be the target of SARS-CoV-2 in the liver. Nevertheless, ALP was not usually as elevated in our or other studies as it might be expected [10, 24]. A proposed mechanism that has gained popularity based on preliminary data includes the cytokine storm associated with systemic inflammatory response syndrome, especially in patients with severe COVID-19 infection [19, 25]. Other explanations put forward include ischemia and reperfusion injury, drug toxicity, and the exacerbation of preexisting chronic liver diseases [24]. However, we did not find a greater requirement for vasoactive drugs or mechanical ventilation in patients with ALEx2 at admission or during hospitalization. Moreover, similar to other studies, no differences were observed between patients with or without abnormal LFT related to medications that were indicated prior to admission [20]. Finally, as was suggested by other authors, it is possible that patients with ALEx2 might present a higher percentage of undiagnosed chronic liver disease [10]. Prior studies showed that patients with underlying liver disease have a higher percentage of liver test abnormalities and decompensated cirrhosis, which were associated with severe COVID-19 [18, 26].

The mortality observed in this series was higher than that reported by others, with about 40% of the fatalities occurring within the first 2 weeks of hospitalization [18]. Compared with another study from Latin America that included more than 1600 patients, the requirement for mechanical ventilation, the need for vasopressors, and the overall mortality were higher in our study, indicating that more severe patients were included [16]. These differences are likely related to the fact that asymptomatic patients with SARS-CoV-2 infection or patients admitted for other causes with COVID-19 diagnosis during hospitalization were excluded in the present study. Another possibility to explain the differences encountered is that we included older patients with a higher rate of comorbidities [16]. Finally, a great proportion of patients were included in our study at the peak of the pandemic in their respective countries, when a higher percentage of less severely ill patients were probably managed on an outpatient basis according to the resources available in each center.

We found cardiovascular comorbidity, active smoking, WBC count at 1 week from admission, and de novo-ALEx2 to be independent risk factors for overall mortality. Cardiovascular comorbidity has been extensively evaluated and has been uniformly identified as one of the main risk factors for mortality associated with COVID-19 [3, 4]. The presence of active smoking at admission has already been indicated as a risk factor as well [27]. Furthermore, active smoking could be a marker of undiagnosed chronic lung disease, a known risk factor associated with severity of infection and death in patients with COVID-19 [3]. WBC count at one week of hospitalization has not been extensively evaluated. A prior study found that elevated WBC count at 1 week of hospitalization was more common in patients with severe COVID-19 compared to nonseverely ill patients [28]. Although this laboratory value may be influenced by several variables as the use of drugs as corticosteroids and the presence of other infections, we found that WBC at one week of hospitalization was an independent predictor of COVID-19-associated death. A multicenter study found that patients of Hispanic ethnicity had a higher risk for severe COVID-19 compared with non-Hispanic whites, even after adjusting for age and comorbidities [26]. The influence of ethnicity was not evaluated in our study, as all the participating centers are from South America, and Hispanic ethnicity was predominant.

The present study has some limitations. Similar to other retrospective studies, there is a possibility of a selection bias leading to the inclusion of more severe cases. Furthermore, the number of patients included in the present study was lower than in other series, which might have limited some of the findings. In addition, autoimmune liver diseases were ruled out mainly by history and a small proportion of patients had autoantibodies assessed prior or during hospitalization, a limitation associated with the retrospective design of the study. Nevertheless, it should be highlighted that the study cohort is representative of 5 countries from South America, one of the regions most severely affected by the pandemic and for which clinical data in COVID-19 are still lacking in medical literature. Another limitation of this study is that medications were not assessed during hospitalization due to the difficulty of evaluating overlapping therapies used for patients with COVID-19. However, considering that the treatment protocols of each center and country were different, it is unlikely that treatments during hospitalization would uniformly affect LFT in all centers.

In summary, in this multicenter study performed in South America, we found that a moderate alteration in LFT at admission was an independent risk factor for short-term mortality in patients with COVID-19. In addition, we showed that de novo-ALEx2 at one week of admission was associated with overall mortality in patients hospitalized for COVID-19. Future prospective studies are necessary to validate the role of LFT alteration during specific periods of hospitalization, in order to identify possible modifications in the assessment of the prognosis of patients admitted with COVID-19.

## Figures and Tables

**Table 1:** Clinical and laboratory characteristics of patients hospitalized for COVID-19 at the moment of admission.

Variable	Total (*n* = 298)	No ALEx2 (*n* = 211)	ALEx2 (*n* = 87)	*P* value
Age (years), mean (SD)	59.7 (15.7)	60.3 (16.4)	58.2 (14)	0.26
Gender (male), *n* (%)	178 (59.7)	114 (54)	64 (73.6)	0.002
Comorbidities, *n* (%)	220 (73.8)	163 (77.3)	57 (65.5)	0.036
Cardiovascular, *n* (%)	70 (23.5)	52 (24.6)	18 (20.7)	0.46
Pulmonary, *n* (%)	34 (11.4)	27 (12.8)	7 (8)	0.24
Kidney, *n* (%)	22 (7.4)	16 (7.6)	6 (6.9)	0.84
Obesity (BMI>35), *n* (%)	56 (18.8)	41 (19.4)	15 (17.2)	0.66
Diabetes, *n* (%)	42 (14.1)	30 (14.2)	12 (13.8)	0.92
Immunosuppression, *n* (%)	15 (5)	10 (4.7)	5 (5.7)	0.72
Chronic liver disease, *n* (%)	21 (7)	11 (5.2)	10 (11.5)	0.054
NAFLD, *n* (%)	17 (5.7)	9 (4.3)	8 (9.2)	0.095
Alcoholic, *n* (%)	3 (1)	2 (0.9)	1 (1.1)	0.87
Hepatitis C, *n* (%)	2 (0.7)	2 (0.9)	0 (0)	0.36
Cirrhosis, *n* (%)	3 (1)	1 (0.5)	2 (2.3)	0.43
Medications at admission, *n* (%)	148 (50)	106 (50.7)	42 (48.2)	0.70
NSAID, *n* (%)	25 (8.4)	19 (9)	6 (6.9)	0.55
Statins, *n* (%)	22 (7.4)	18 (8.5)	4 (4.6)	0.24
Antibiotics, *n* (%)	101 (33.9)	69 (32.7)	32 (36.8)	0.5
Acetaminophen, *n* (%)	48 (16.1)	35 (16.6)	13 (14.9)	0.73
Other, *n* (%)	64 (21.5)	46 (21.8)	18 (20.7)	0.83
Active smoker, *n* (%)	39 (14.1)	30 (15.1)	9 (11.5)	0.45
Symptoms				
Fever (38^o^C), *n* (%)	195 (65.4)	139 (65.9)	56 (64.4)	0.8
Dyspnea, *n* (%)	219 (73.5)	152 (72)	67 (77)	0.38
Cough, *n* (%)	177 (59.4)	119 (56.4)	58 (66.7)	0.1
Diarrhea, *n* (%)	50 (16.8)	34 (16.1)	16 (18.4)	0.63
Nausea/vomiting, *n* (%)	22 (7.4)	17 (8.1)	5 (5.7)	0.49
Abdominal pain, *n* (%)	23 (7.7)	17 (8.1)	6 (6.9)	0.73
Others, *n* (%)	138 (46.3)	103 (48.8)	35 (40.2)	0.18
Time from symptoms to hospitalization (days), median (IQR)	7 (4–10)	6 (3–9)	7 (5–10)	0.017
Hemoglobin (g/L), median (IQR)	13.9 (12.4–15)	13.7 (12.3–15)	14.2 (12.9–15.1)	0.14
WBC (x10^9^/L), median (IQR)	7.8 (5.6–11.4)	7.8 (5.4–11.2)	7.7 (6.2–12.5)	0.57
Neutrophils/lymphocytes ratio	5.9 (3.4–10.6)	5.6 (3.2–10)	7.4 (4.3–12.1)	0.036
Platelets (x10^9^/L), median (IQR)	221 (164–280)	226.5 (166–294)	201.5 (145.8–265.3)	0.033
Glucose at admission (mg/dL), median (IQR)	123 (105–160.3)	125 (106–163)	119 (103–152)	0.51
C-reactive protein (mg/L), median (IQR)	16.3 (6.3–82)	15 (5.5–79)	18.4 (6.9–87.5)	0.44
Procalcitonin (mg/L), median (IQR)	0.15 (0.08–0.35)	0.16 (0.09–0.48)	0.15 (0.07–0.3)	0.69
INR, median (IQR)	1.07 (1–1.17)	1.06 (1–1.18)	1.07(1–1.17)	0.85
D-dimer (mg/L), median (IQR)	0.71 (0.33–2.12)	0.64 (0.29–1.8)	0.75 (0.42–4.4)	0.08
Ferritin (mg/L), median (IQR)	842 (509–1644)	800 (446–1262)	1127 (618–2522)	0.004
LDH (U/L), median (IQR)	396 (294–539)	371 (289–514)	454 (321–594)	0.022
AST (U/L), median (IQR)	41 (28–63)	35.5 (24.5–48)	74 (49–99)	<0.001
ALT (U/L), median (IQR)	42 (27–68.5)	35 (23–49)	93 (61–127)	<0.001
GGT (U/L), median (IQR)	50.5 (34–100.5)	43 (28–56)	142 (105–209)	<0.001
ALP (U/L), median (IQR)	86.5 (69–119)	80 (65.3–104.3)	108.5 (78–152.8)	<0.001
Total bilirubin (mg/dL), median (IQR)	0.53 (0.39–0.87)	0.49 (0.31–0.71)	0.63 (0.41–1.2)	<0.001
Direct bilirubin (mg/dL), median (IQR)	0.29 (0.19–0.47)	0.22 (0.17–0.38)	0.39 (0.23–0.7)	<0.001
Albumin (g/dL), median (IQR)	3.7 (3.2–4.1)	3.8 (3.3–4.1)	3.6 (2.9-4-2)	0.29
Serum creatinine (mg/dL), median (IQR)	0.87 (0.69–1.09)	0.85 (0.69–1.08)	0.87 (0.69–1.1)	0.97
Sodium (mEq/L), median (IQR)	137 (134–140)	137 (134–140)	137 (134–140)	0.98
Potassium (MEq/L), median (IQR)	4.03 (3.7–4.43)	4 (3.7–4.4)	4.18 (3.7–4.6)	0.31
Radiological findings at admission				
Unilateral infiltrate, *n* (%)	28 (9.4)	25 (11.8)	3 (3.4)	0.024
Bilateral infiltrate/consolidation, *n* (%)	247 (82.9)	168 (79.6)	79 (90.8)	0.02

ALEx2, abnormal liver enzymes >2 times over the upper limit of normal; BMI, body mass index; NAFLD, nonalcoholic fatty liver disease; NSAID, nonsteroidal anti-inflammatory drug; IQR, interquartile range; WBC, white blood cells; INR, international normalized ratio; LDH, lactate dehydrogenase; AST, aspartate aminotransferase; ALT, alanine aminotransferase; GGT, gamma-glutamyl transferase; ALP, alkaline phosphatase.

**Table 2:** Clinical course of patients hospitalized for COVID-19.

Variable	No ALEx2 (*n* = 211)	ALEx2 (*n* = 87)	*P* value
Transfer to ICU, *n* (%)	99 (47.1)	38 (43.7)	0.59
Vasopressors use, *n* (%)	45 (21.8)	20 (23.3)	0.8
Need for mechanical ventilation, *n* (%)	72 (34.1)	27 (31)	0.61
Length of mechanical ventilation (days), median (IQR)	15 (8–24.8)	13 (7.5–16.5)	0.11
Length of ICU admission (days), median (IQR)	13 (6–23.5)	12 (5–16.8)	0.13
Length of hospitalization (days), median (IQR)	14 (7–23.5)	11 (7–20)	0.055
Mortality			
Days 0–7, *n* (%)	6 (2.8)	8 (9.2)	0.031
Days 8–14, *n* (%)	9 (4.4)	4 (5.1)	0.85
Days 15–21, *n* (%)	16 (8.2)	7 (9.4)	0.91
Days 22–28, *n* (%)	2 (1.1)	1 (1.5)	0.63
Days 29–60, *n* (%)	12 (6.7)	1 (1.5)	0.15
Overall, *n* (%)	45 (21.5)	21 (24.1)	0.62

ALEx2, abnormal liver enzymes >2 times over the upper limit of normal; ICU, intensive care unit; IQR, interquartile range.

**Table 3:** Distribution of abnormal liver enzymes at admission and at 1 week of hospitalization.

Liver enzyme	Patients with ALEx2 at admission (*n* = 87)	Patients with de novo-ALEx2 (*n* = 84)
AST > 2UNL, *n* (%)	59 (67.8)	67 (79.8)
ALT >2 UNL, *n* (%)	46 (52.9)	45 (53.6)
TBil >2 UNL, *n* (%)	6 (6.9)	8 (9.5)
ALP >2 UNL, *n* (%)	8 (9.2)	11 (13.1)
GGT >2 UNL, *n* (%)	32 (36.2)	13 (15.5)

ALEx2, abnormal liver enzymes >2 times over the upper limit of normal; AST, aspartate aminotransferase; ALT, alanine aminotransferase; TBil, total bilirubin; GGT, gamma-glutamyl transferase; ALP, alkaline phosphatase.

**Table 4:** Multivariate logistic regression evaluating risk factors for death associated with COVID-19 during different periods of hospitalization.

Variable	First week (days 0–7)		Second week (days 8–14)		Overall hospitalization	
	Univariate	Multivariate	Univariate	Multivariate	Univariate	Multivariate
Age (years)	1.04 (1.002–1.08)	—	1.08 (1.04–1.12)	—	1.05 (1.03–1.07)	—
Gender (male)	2.57 (0.7–9.41)	—	2.1 (0.7–6.7)	—	1.57 (0.86–2.85)	—
Cardiovascular comorbidity	4.77 (1.59–12.28)	8.65 (2.37–31.62)	6.17 (2.16–17.7)	9.53 (2.17–41.9)	3.75 (2.08–6.75)	3.1 (1.14–8.45)
Obesity (BMI>35)	1.06 (1.03–1.1)	—	3.63 (0.47–28.1)	—	2.87 (1.17–7.02)	—
Diabetes	1.02 (0.2–4.71)	—	0.86 (0.19–3.9)	—	1.1 (0.49–2.43)	—
Chronic liver disease	4.3 (1.03–15.75)	7.17 (1.51–34.16)	1.76 (0.48–6.4)	—	4.25 (1.72–10.5)	4.25 (0.95–19)
Time from symptoms to hospitalization (days)	0.99 (0.97–1.02)	—	0.98 (0.96–0.99)	—	0.99 (0.97–1.01)	—
Active smoker	11.35 (0.29–6.62)	—	1.44 (0.39–5.31)	—	2.7 (1.3–5.46)	6.45 (1.98–21)
WBC count (x109/L) on admission	1.15 (1.07–1.24)	1.17 (1.08–1.28)	1 (0.91–1.1)	—	1.07 (1.02–1.12)	—
WBC count (x109/L) at 1 week from admission	—	—	1.1 (0.95–1.17)	—	1.1 (1.04–1.18)	1.13 (1.04–1.23)
ALEx2 at admission	4.75 (1.55–14.62)	3.55 (1.05–12.05)	0.8 (0.25–2.5)	—	1.11 (0.62–2)	—
De novo-ALEx2 at 1 week after admission	—	—	6.3 (1.53–25.9)	6.09 (1.28–29)	2.79 (1.21–6.43)	2.93 (1.05–8.19)
Persistent-ALEx2 at 1 week after admission	—	—	9.57 (1.12–81.9)	4.98 (0.53–46.7)	3.24 (1.18–8.87)	3.73 (0.91–15.37)
Neutrophils/lymphocytes ratio	1.05 (1.01–1.08)	—	1 (0.95–1.05)	—	1.02 (0.99–1.05)	—
C-reactive protein (mg/L)	1 (0.99–1.003)	—	1 (0.99–1.01)	—	1.004 (1.001–1.006)	—
Procalcitonin (mg/L)	1.003 (0.91–1.09)	—	0.99 (0.91–1.09)	—	1.005 (0.97–1.05)	—
Serum creatinine (mg/dL)	1.22 (0.96–1.57)	—	1.37 (1.09–1.71)	—	1.24 (1.01–1.5)	—
Bilateral consolidation at admission	0.75 (0.2–2.77)	—	0.31 (0.04–2.4)	—	2.15 (1.12–4.13)	—

ALEx2, abnormal liver enzymes >2 times over the upper limit of normal; BMI, body mass index; WBC, white blood cells.

## Data Availability

The database used to support the findings of this study is available from the corresponding author upon request.
